# Associations of cerebrospinal fluid amyloidogenic nanoplaques with cytokines in Alzheimer’s disease

**DOI:** 10.1186/s40035-021-00244-3

**Published:** 2021-06-08

**Authors:** Mari Aksnes, Hans Christian D. Aass, Ann Tiiman, Trine Holt Edwin, Lars Terenius, Nenad Bogdanović, Vladana Vukojević, Anne-Brita Knapskog

**Affiliations:** 1 Department of Geriatric Medicine, Institute of Clinical Medicine, Faculty of Medicine, University of Oslo, Oslo, Norway; 2 Department of Medical Biochemistry, Oslo University Hospital, Oslo, Norway; 3 Department of Clinical Neurosciences (CNS), Center for Molecular Medicine CMM L8:01, Karolinska Institutet, Stockholm, Sweden; 4 Department of Geriatric Medicine, The Memory Clinic, Oslo University Hospital, Oslo, Norway; 5 Institute of Health and Society, Faculty of Medicine, University of Oslo, Oslo, Norway; 6 Norwegian National Advisory Unit on Ageing and Health, Vestfold Hospital Trust, Tønsberg, Norway; 7 Department of Neurobiology, Care Science and Society (NVS), Division of Clinical Geriatrics, Karolinska Institutet, Huddinge, Sweden

**Keywords:** Alzheimer's disease, Amyloid, Amyloid beta peptides, Amyloidogenic proteins, Biomarkers, Cerebrospinal fluid, Cytokines, Inflammation, Fluorescence correlation spectroscopy, Thioflavin T

## Abstract

**Background:**

The aggregation of amyloid β (Aβ) is central in the pathogenesis of Alzheimer’s disease (AD). Recently it has been shown that specifically, larger, Thioflavin T-binding Aβ aggregates are associated with increased neuroinflammation and cytokine release. This study was aimed to quantify fibrillary amyloid aggregates, so-called nanoplaques, and investigate their relationship with cytokines in the cerebrospinal fluid (CSF).

**Methods:**

CSF was collected from 111 patients assessed for cognitive complaints at the Oslo University Hospital Memory Clinic. The patients were grouped based on their amyloid status. The CSF nanoplaque concentration was quantified with the Thioflavin T-fluorescence correlation spectroscopy (ThT-FCS) assay. The levels of nine cytokines (eotaxin-1, granulocyte stimulating factor, interleukin [IL]-6, IL-7, IL-8, monocyte chemoattractant protein-1, gamma-induced protein 10, macrophage inflammatory protein [MIP]-1α, and MIP-1β) were quantified with a magnetic bead-based multiplex assay and read on a Luminex IS 200 instrument.

**Results:**

There were 49 amyloid-negative and 62 amyloid-positive patients in the cohort; none of the cytokines differed significantly between the amyloid groups. The increased nanoplaque levels were associated with levels of MIP-1β below the lower limit of quantification, and with decreased levels of MIP-1α and IL-8. The associations remained significant when adjusted for age, sex, cognitive function, apolipoprotein ε4 status and CSF core biomarker levels.

**Conclusion:**

The cytokine levels were not associated with amyloid status in this cohort. The nanoplaque levels were negatively associated with MIP-1β, MIP-1α and IL-8, which is in line with recent findings suggesting that the upregulation of some cytokine markers has a protective role and is negatively associated with AD progression.

## Background

Alzheimer’s disease (AD) is the leading cause of dementia globally. This neurodegenerative disorder is characterised by extracellular amyloid β (Aβ) deposits, intracellular tau neurofibrillary tangles and pervasive synaptic loss [1]. These neuropathological changes precede the onset of clinical dementia by years or decades [2, 3]. The primary cause of sporadic AD is unknown, but it is hypothesised that the accumulation of Aβ aggregates is the initial trigger [4]. In addition, increasing evidence has linked neuroinflammation to AD pathogenesis, particularly amyloid pathology [5]. Astrocytes and microglia, immune cells of the brain, cluster around Aβ aggregates.

Activated astrocytes and microglia release cytokines, which are signalling proteins with both pro- and anti-inflammatory effects [5]. In AD, this causes a self-propagating inflammatory cycle where Aβ aggregates continually activate microglia, provoking the release of cytokines, which in turn affect Aβ production and aggravate the plaque formation [5, 6]. As such, cytokines are upregulated early in the Aβ-induced inflammatory process, and are interesting biomarkers for AD [7]. Several cytokines have been found to differentiate AD patients from patients with mild cognitive impairment (MCI) or cognitively unimpaired controls [8], but inconsistent results are common. In addition, investigations of the relationship between established markers of AD neuropathology such as cerebrospinal fluid (CSF) Aβ_42_ and cytokines have resulted in inconsistent findings [9–11].

Importantly, Aβ aggregates are not uniform, and range from small oligomers to larger protofibrils and fibrils [12, 13]. The aggregates vary in size and molecular structure, which results in different mechanisms of toxicity. Recently, cell culture investigations have shown that while smaller soluble aggregates exert neurotoxicity by increasing the cell membrane permeability, larger protofibrils that can bind Thioflavin T increase neuroinflammation and cytokine release from glial cells [14, 15]. Moreover, such fibrillar aggregates appear to contribute to neuroinflammation by increasing the blood-brain barrier permeability in vitro [16]. Of note, it has been shown that longer, inflammation-inducing amyloid fibrils are increased in AD CSF compared to the CSF from MCI patients and healthy controls [14]. However, the relationship between these fibrils and other biomarkers has not been established. It would be of interest to extend research on fibrillary amyloid aggregates and inflammatory markers in cell cultures to clinical samples. This highlights the importance of quantifying different Aβ species as AD biomarkers. Indeed, whereas the established enzyme-linked immunosorbent Aβ_42_ assays primarily recognise monomeric Aβ, the Thioflavin T-fluorescence correlation spectroscopy (ThT-FCS) assay quantifies structured amyloidogenic aggregates, so-called nanoplaques, with high specificity [17–19]. As such, the relationship between ThT-FCS-labelled nanoplaques and markers of neuroinflammation, such as cytokines, should be explored.

In the current study, we set out to investigate the relationship between CSF nanoplaque levels and nine cytokines in a memory clinic cohort, including both patients with AD pathology and patients with non-AD disorders.

## Methods

### Memory clinic cohort

One hundred and eleven patients from the Norwegian Registry of Persons Assessed for Cognitive Symptoms (NorCog), who were assessed at the Oslo University Hospital Memory Clinic, were included in this study. The patients were included in NorCog between June 2014 and November 2018.

### Clinical assessment

The patients were assessed according to an established research protocol [20]. The patients completed a battery of standardised cognitive tests [20], including the Mini-Mental State Examination (MMSE), and also underwent a physical examination, including blood sampling, lumbar puncture, apolipoprotein E (*APOE*) genotyping, magnetic resonance imaging (MRI, *n* = 107), ^18^F-FDG positron emission tomography (PET, *n* = 65) and ^18^F-flutemetamol PET (*n* = 54) brain scans. The core CSF biomarkers for AD were analysed for all patients (*n* = 111) at the Department of Interdisciplinary Laboratory Medicine and Medical Biochemistry at Akershus University Hospital with enzyme-linked immunosorbent assays using the Innotest kit (Innogenetics, Ghent, Belgium). Cognitive and functional impairment on the Clinical Dementia Rating (CDR) scale were scored *post-hoc* by experienced CDR raters (ABK and THE). The CDR sum-of-boxes (CDR-SOB), which has been found to accurately separate MCI and dementia [21], was used in the analyses.

Clinical diagnoses were made *post-hoc* by experienced memory clinicians (ABK and THE). Subjective cognitive decline (SCD) was diagnosed according to the Subjective Cognitive Decline Initiative-criteria [22], whilst MCI and dementia were diagnosed using the core clinical criteria of the National Institute of Aging and the Alzheimer’s Association (NIA-AA) [23, 24]. Clinical diagnoses of MCI-AD, AD-dementia and AD-dementia aetiologically mixed presentation, collectively Alzheimer’s clinical syndrome, were made following the NIA-AA diagnostic criteria [23, 24]. These diagnoses were primarily based on clinical presentation, however, in the case of MCI patients with atypical or mixed presentations, biomarker data were consulted and patients with negative biomarker data were considered non-AD. Together with the SCD patients, the patients diagnosed with vascular dementia [25], frontotemporal dementia [26], and MCI not caused by AD were considered as clinically non-AD.

### AT(N) classification

In line with the 2018 NIA-AA research framework, patients were categorised based on three groups of biomarker data: A – reflecting Aβ pathology, T – reflecting tau pathology and N – reflecting neurodegeneration [27]. The patients were classified according to the AT(N) status based on all available biomarkers: CSF Aβ_42_ and/or ^18^F-flutemetamol results for A; CSF phosphorylated tau (P-tau) for T; and CSF total tau (T-tau), ^18^F-FDG PET and/or MRI results for N. The ^18^F-flutemetamol PET scans were visually classified as amyloid-positive or negative according to a validated electronic image reader programme [28]. The FDG PET and MRI scans were also visually classified for signs of neurodegeneration [29, 30]. For the CSF biomarkers, the following cut-off values were applied for a normal test: Aβ_42_ > 700 pg/ml, P-tau < 80 pg/ml, and T-tau < 300 pg/ml for those < 50 years, < 450 pg/ml for those aged 50–70 years and < 500 pg/ml for those > 70 years [31]. In the case of the presence of disparity among the biomarkers in one group, the patient was classified as positive. In the statistical analyses, patients were categorised based exclusively on amyloid status (A+/A- groups).

### Analysis of CSF nanoplaque levels

The ThT-FCS assay procedure has been described in detail by Tiiman et al. [19]. Briefly, 1.6 μL of 2.5 mM ThT in deionised water was mixed with 200 μL of CSF. Fluctuations in fluorescence intensity were recorded in duplicates using the ConfoCor3 system (Carl Zeiss, Jena, Germany) [32]. Signals were collected in 30 series of 10 × 10 s measurements (total measurement time 3000 s). Automated analysis of fluorescence intensity fluctuations and detection of bursts in fluorescence intensity that reflect the passage of ThT-positive nanoplaques through the observation volume element, were performed offline using a dedicated software written in MATLAB (The MathWorks, Inc., Natick, MA) by Tiiman et al. [19]. A burst was identified and denoted a “single event” if the increase in fluorescence intensity was at least five times larger than the standard deviation of the whole time series. The total number of single events per hour, i.e. the frequency of single events per hour (*f*seo), directly reflects the concentration of nanoplaques in the CSF.

### Analysis of cytokines

All CSF samples underwent analysis of cytokines using a custom-made nine-plex kit (Cat No. 12014058, Bio-Rad Laboratories, Hercules, CA) containing eotaxin-1, granulocyte colony-stimulating factor (G-CSF), interleukin (IL)-6, IL-7, IL-8, interferon gamma-induced protein 10 (IP-10), monocyte chemoattractant protein 1 (MCP-1), macrophage inflammatory protein (MIP)-1α, and MIP-1β. The nine cytokines were selected based on a screening of a representative set of samples with the human cytokine 27 plex kit (Cat No. M500KCAF0Y, Bio-Rad Laboratories). A 10% bovine serum albumin (Cat No. A5403-50G, lot SLBL9495V, Sigma Aldrich, St. Louis, MO) solution in PBS (pH 7.4, Gibco Cat No. 10010–015, lot 2062123, Thermo Fisher Scientific, Waltham, MA) was added to all CSF samples to a final concentration of 0.5% and vortexed. Then the samples were centrifuged at 10 000×g for 10 min at 4 °C and 50 μL of the supernatant was loaded onto the assay plate. The CSF cytokine levels were measured on a Luminex IS 200 instrument (Bio-Rad). An in-house control was used to observe both intra percentage coefficient of variation and longitudinal (inter) percentage coefficient of variation. Cytokine concentrations below the lower limit of quantification (LLOQ) or above the upper limit of quantification extrapolated by the analysis software were also included in the statistical analysis.

### Statistical analysis

Group differences were assessed by parametric (*t*-test) or non-parametric (Mann-Whitney test) test depending on the data distribution. As the distributions of CSF nanoplaque levels and most of the cytokines were markedly right-skewed, the results are presented as medians and quartiles. The correlation analyses were reported with Spearman’s ρ correlation coefficient. Bonferroni-corrected *P-*values are presented for the correlations between nanoplaque levels and cytokines. Significant associations between nanoplaque levels and cytokines were further explored in multiple linear regression analyses adjusted for sex, age, CDR-SOB and *APOE* ε4 carrier status (Model 1), and biomarkers CSF Aβ_42_ and CSF P-tau (Model 2). The natural logarithm transformed nanoplaque variable log (*f*seo) was used in the regression analysis in order to achieve normally distributed standardised residuals. Statistical analyses were conducted with STATA 15.1 (StataCorp, College Station, TX) and R 3.4.4 (R Foundation for Statistical Computing, Vienna, Austria).

## Results

Patient characteristics are presented in Table 1. The A+ and A- groups differed significantly in all variables except the years of education (*P* = 0.34). In the A+ group, 28 patients (45%) were T+ and 58 patients (94%) were N+, whereas in the A- group, 8 patients (16%) were T+ and 31 patients (63%) were N+. The clinical diagnosis was consistent with the amyloid status: 89% of the A+ patients had Alzheimer’s clinical syndrome, and 84% of the A- patients had clinical non-AD.

**Table 1:** Demographic and clinical characteristics for all patients and by amyloid group

	All (***n*** = 111)	A- (***n*** = 49)	A+ (***n*** = 62)	***P***
Number of women/men	57/54	20/29	37/25	0.05
Age in years	65.0 (8.4)	61.4 (8.9)	67.8 (6.8)	< 0.01
Years of education	13.8 (3.6)	14.2 (3.4)	13.5 (3.7)	0.34
MMSE*	25.7 (4.1)	27.1 (3.0)	24.6 (4.4)	< 0.01
CDR-SOB*	3.3 (2.4)	2.6 (2.3)	3.9 (2.3)	< 0.01
*APOE* ε4*, *n* (%)	63 (57.8)	17 (35.4)	46 (75.4)	< 0.01
Clinical diagnosis				< 0.01
Clinical non-AD	48 (43.2)	41 (83.7)	7 (11.3)	
Alzheimer’s clinical syndrome	63 (56.8)	8 (16.3)	55 (88.7)	
Stage, *n* (%)				< 0.01
SCD	11 (9.9)	9 (18.4)	2 (3.2)	
MCI	42 (37.8)	27 (55.1)	15 (24.2)	
Dementia	58 (52.3)	13 (26.5)	45 (72.6)	
CSF Aβ_42_ pg/ml	777.3 (353.8)	1110.6 (231.1)	513.9 (154.8)	< 0.01
CSF T-tau pg/ml	500.2 (367.9)	336.9 (167.5)	629.2 (428.5)	< 0.01
CSF P-tau pg/ml	71.7 (39.9)	55.7 (22.3)	84.3 (46.0)	< 0.01
CSF nanoplaques, median (Q1;Q3)	15 (11.4; 21.6)	13.2 (10.8; 17.4)	18 (12; 24.6)	0.02^†^

Data are given as mean (standard deviation) and *P* values are for two-tailed *t*-tests (continous variables) or *χ*^2^-tests (categorical variables) comparing amyloid groups, unless otherwise indicated

*Aβ* amyloid-β, *AD* Alzheimer’s disease, *APOE* apolipoprotein E, *CDR-SOB* Clinical Dementia Rating Scale Sum of Boxes, *CSF* cerebrospinal fluid, *MCI* mild cognitive impairment, *MMSE* Mini-Mental State Examination, *n* number of patients, *P-tau* phosphorylated tau, *Q* quartile, *SCD* subjective cognitive decline, *T-tau* total tau

*MMSE, *n* = 104; CDR-SOB, *n* = 108; *APOE*, *n* = 109, † *P-*value for Mann-Whitney U test

The CSF Aβ_42_ levels were negatively associated with the levels of CSF nanoplaques (*ρ* = − 0.20, *P* = 0.04), CSF T-tau (*ρ* = − 0.39, *P <* 0.001) and CSF P-tau (*ρ* = − 0.30, *P* = 0.001). Moreover, the levels of CSF P-tau and CSF T-tau were strongly correlated with each other (*ρ* = 0.94, *P* <  0.001). The CSF nanoplaque levels were not associated with CSF P-tau (*ρ* = 0.001) or CSF T-tau (*ρ* = − 0.01), *P* > 0.05.

### Detectability of cytokines

The markers eotaxin-1, IL-8, MCP-1 and IP-10 were detectable in all patient CSF samples. In a minority of samples, the levels of G-CSF (*n* = 4, 3.6%), IL-6 (*n* = 1, 0.9%) and MIP-1α (*n* = 1, 0.9%) were below the LLOQ, and therefore included as zero. In the majority of samples, the levels of MIP-1β (*n* = 77, 68.8%) and IL-7 (*n* = 71, 63.4%) were below the LLOQ, so MIP-1β and IL-7 were dichotomised as detectable/not detectable.

### Cytokine levels between amyloid groups

The CSF levels of cytokines are presented in Table 2. There were no significant differences in the levels of cytokines or the detectability of the dichotomised markers between the amyloid groups (*P >* 0.05).

**Table 2:** CSF cytokine levels for all patients and by amyloid group

	All (***n*** = 111)	A- (***n*** = 49)	A+ (***n*** = 62)	***P***
**Continuous markers (pg/mL), median (Q1; Q3)**^**a**^				
Eotaxin-1	3.95 (3.27; 4.72)	3.93 (3.48; 4.72)	3.97 (3.16; 4.49)	0.73
G-CSF	15.75 (6.37; 27.94)	11.79 (5.16; 29.2)	16.24 (8.33; 25.91)	0.46
IL-6	1.51 (1.00; 2.31)	1.44 (1.09; 2.45)	1.54 (1.00; 2.06)	0.58
IL-8	20.59 (17.58; 25.44)	20.58 (18.11; 25.12)	20.66 (17.33; 25.44)	0.54
IP-10	1740 (1333; 2196)	1845 (1333; 2205)	1652 (1342; 2118)	0.49
MCP-1	153.87 (127.44; 177.55)	142.39 (127.44; 177.34)	154.64 (129.57; 177.72)	0.61
MIP-1α	0.33 (0.2; 0.42)	0.31 (0.17; 0.40)	0.34 (0.23; 0.49)	0.10
**Dichotomised markers,** ***n*** **(% detected)**^**b**^				
MIP-1β	34 (30.63)	16 (32.65)	18 (29.03)	0.68
IL-7	40 (36.04)	17 (34.69)	23 (37.10)	0.79

*G-CSF* granulocyte colony-stimulating factor, *IL* interleukin, *IP* interferon gamma-induced protein, *MCP* monocyte chemoattractant protein, *MIP* macrophage inflammatory protein, *n* number of patients, *Q* quartile

^a^Two-tailed Mann-Whitney U test was used for group comparisons, ^b^*χ*^2^-test was used for group comparisons

### Cytokine and nanoplaque levels

**Fig. 1:**
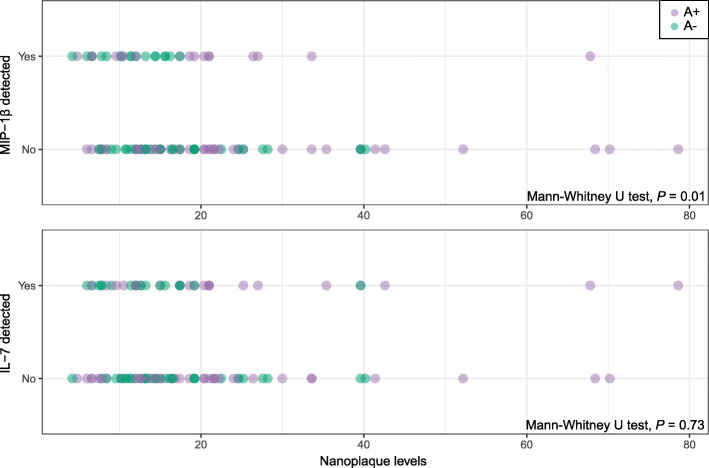
Distribution of nanoplaque levels in relation to MIP-1β and IL-7 detectability. Purple dots indicate A+ patients, green dots indicate A- patients; darker colours indicate overlapping values

The distributions of nanoplaque levels in the patients with non-detectable/detectable levels of MIP-1β and IL-7 are presented in Fig. 1. The nanoplaque levels were increased among patients with non-detectable levels of MIP-1β (median = 16.5, inter-quartile range: 12.6; 24) compared to those with detectable levels of MIP-1β (median = 12.6, inter-quartile range: 9.6; 18.6, *P* = 0.01). There was no difference in the nanoplaque levels between those with non-detectable levels of IL-7 (median = 15, inter-quartile range: 11.4; 22.2) and those with detectable levels of IL-7 (median = 15.3, inter-quartile range: 10.95; 21, *P* = 0.73).

**Fig. 2:**
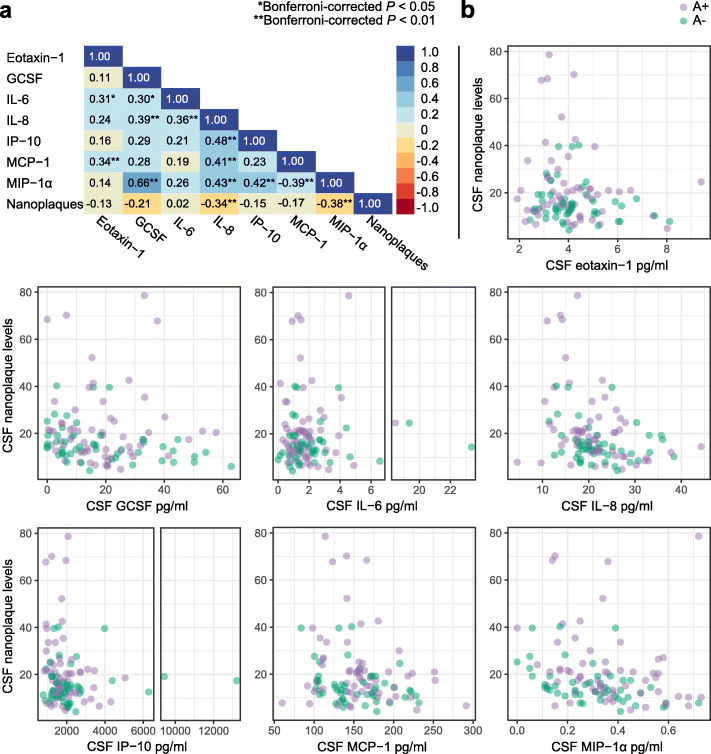
The associations between CSF nanoplaques and cytokine levels. **a** Spearman correlation matrix for all cytokines and CSF nanoplaque levels. **b** Scatter plots of CSF nanoplaque levels versus each of the cytokines. Note the broken abscissa for IL-6 (middle) and IP-10 (bottom left). Purple dots indicate A+ patients, green dots indicate A- patients; darker colours indicate overlapping values. There was no significant difference in cytokine levels between the A+ and A- patients

The nanoplaque levels were negatively associated with IL-8 (*ρ* = − 0.34, *P =* 0.007) and MIP-1α levels (*ρ* = − 0.38, *P* = 0.001), but not associated with any of the other cytokines (Fig. 2).

The negative association between IL-8 and nanoplaque levels remained significant when adjusting for the covariates sex, age, cognitive function and *APOE* ε4 carrier status (*P* = 0.001), and for Aβ_42_ and P-tau levels (*P* = 0.003) in two regression models (Table 3). None of the covariates were significantly associated with IL-8.

**Table 3:** Adjusted associations between nanoplaque levels and IL-8 and MIP-1α

	Model 1		Model 2	
	β-coefficient (CI)	*P*	β-coefficient (CI)	*P*
**Dependent variable: IL-8 pg/ml**				
CSF nanoplaques^a^	−3.830 (−6.038; −1.622)	0.001	−3.536 (−5.821; −1.250)	0.003
Male sex	−0.802 (−3.376; 1.773)	0.538	− 1.071 (− 3.682; 1.540)	0.418
Age	−0.054 (− 0.214; 0.106)	0.505	− 0.029 (− 0.203; 0.144)	0.737
CDR-SOB	0.170 (− 0.390; 0.731)	0.548	0.144 (− 0.431; 0.720)	0.620
*APOE* ε4	−0.452 (− 1.818; 0.915)	0.513	− 0.373 (− 1.799; 1.054)	0.605
CSF Aβ_42_ pg/ml			0.002 (−0.002; 0.007)	0.321
CSF P-tau pg/ml			0.018 (−0.015; 0.052)	0.285
**Dependent variable: MIP-1α pg/ml**				
CSF nanoplaques^a^	−0.083 (− 0.133; − 0.034)	0.001	−0.079 (− 0.127; − 0.031)	0.002
Male sex	0.049 (− 0.010; 0.107)	0.101	0.037 (− 0.018; 0.092)	0.180
Age	0.005 (0.002; 0.009)	0.006	0.005 (0.001; 0.009)	0.009
CDR-SOB	0.012 (−0.001; 0.025)	0.064	0.007 (−0.005; 0.019)	0.233
*APOE* ε4	0.030 (−0.001; 0.061)	0.058	0.023 (−0.007; 0.053)	0.128
CSF Aβ_42_ pg/ml			0.000 (−0.000; 0.000)	0.486
CSF P-tau pg/ml			0.001 (0.001; 0.002)	< 0.001

*Aβ* amyloid-β, *APOE* apolipoprotein E, *CI* confidence interval, *CDR-SOB* Clinical Dementia Rating Scale Sum of Boxes, *CSF* cerebrospinal fluid, *f*seo frequency of single event occurrence per hour, *IL-8* interleukin-8, *MIP-1α* macrophage inflammatory protein 1α, *P-tau* phosphorylated tau

^a^Log (*f*seo). The β-coefficient denotes the degree of change in the dependent variable (IL-8 or MIP-1α) for a one-step change in the continuous predictor variables or the presence of the dichotomised predictor variables

Similarly, the negative association between MIP-1α and nanoplaque levels remained significant when adjusting for the covariates sex, age, cognitive function and *APOE* ε4 carrier status (*P* = 0.001) and for Aβ_42_ and P-tau levels (*P* = 0.002) in the two regression models (Table 3). In the first model (Model 1, Table 3), increased age significantly predicted increased MIP-1α levels, with a 0.05 pg/ml increase of MIP-1α levels for each 10-year increase of age (*P* = 0.006). In the second model (Model 2, Table 3), age remained a significant predictor for increased MIP-1α and the increased levels of P-tau were associated with increased levels of MIP-1α, with a 10 pg/ml increase in P-tau predicting a 0.014 pg/ml increase in MIP-1α levels (*P* < 0.001). The decreased nanoplaque levels were the strongest predictor for increased MIP-1α levels in both models.

## Discussion

There has been mounting evidence suggesting that inflammatory mediators released by microglia and astrocytes, such as cytokines, contribute to AD neuropathology [5]. Amyloid pathology has been linked to increased neuroinflammation; in vitro ThT-reactive amyloid aggregates have been specifically shown to induce cytokine release [15]. In this study, we used the ThT-FCS assay to quantify ThT-reactive amyloidogenic aggregates (nanoplaques) in the CSF of memory clinic patients. For the first time, we investigated the association between these nanoplaques and CSF cytokine levels, and had two main findings: first, three cytokines were negatively associated with CSF nanoplaque levels, and second, the CSF cytokine levels did not differ between amyloid-positive and amyloid-negative patients.

It has been hypothesised that larger amyloid protofibrils in the CSF of AD patients cause an increase in inflammation [14]. The nanoplaques quantified by the ThT-FCS assay include both protofibrils and other ThT-reactive amyloid aggregates composed of > 40 monomers. However, in the current study, the CSF nanoplaque levels were negatively associated with the CSF levels of IL-8, MIP-1α and MIP-1β. While we have previously shown that the CSF nanoplaque levels are negatively associated with CSF Aβ_42_ [33], the association between nanoplaques and these cytokines appears independent of the CSF Aβ_42_ and P-tau levels. Although amyloid aggregates have been shown to increase the release of cytokines [14, 15], this finding is in line with recent research showing that the upregulation of several cytokines, including G-CSF and MIP-1β, is negatively associated with AD disease progression and may have a neuroprotective role [34]. As such, it is possible that the higher level of cytokines interferes with the formation of nanoplaques, and that nanoplaque levels increase when cytokine levels are downregulated.

Indeed, the inflammatory activity in AD is complex and may change across disease stages. It has been proposed that there are two peaks of inflammatory (microglial) activity in AD: an early, potentially neuroprotective peak, and a later, neurotoxic peak [35]. In line with the two-peak hypothesis, a recent longitudinal PET study has demonstrated a biphasic trajectory of cortical inflammation in early AD [36]: over a two-year period, microglial activation increased in MCI patients who converted from amyloid-negative to amyloid-positive, but in MCI patients who were amyloid positive at baseline, the cortical inflammation decreased as amyloid levels plateaued. Furthermore, this study also showed that inflammation increased with the increasing tangle load in amyloid-positive patients with tau pathology at baseline [36]. As the nanoplaque levels are a measure of amyloid aggregation that is not correlated with amyloid-PET [17], it would be of interest to follow the relationship between nanoplaques and inflammatory markers in longitudinal studies.

In the central nervous system, cytokines can be produced by microglia, astrocytes and neurons. In vitro*,* microglia, astrocytes and neurons have been shown to release IL-8 in response to other pro-inflammatory cytokines such as IL-1β [37, 38]. Similarly, MIP-1α and MIP-1β appear to be expressed by both microglia and astrocytes. In microglial cell cultures, expression of MIP-1α and MIP-1β is increased in response to fibrillary Aβ [39]. However, in animal models of AD, MIP-1α production appears to be driven by astrocytes [40]. In the AD brain, MIP-1β is expressed by a subpopulation of astrocytes (together with IP-10) [41]. Moreover, eotaxin-1 is released by astrocytes [42]; G-CSF is expressed by neurons [43], whereas IL-6 and IL-7 appear to be primarily expressed by microglia [44, 45]. As a consequence of these complex interactions in neuroinflammation, it is uncertain what proportion of the measured cytokines is derived from which source.

Inflammatory markers have been extensively studied as biomarkers for AD, both in the CSF and in peripheral samples [46, 47]. A recent meta-analysis of 170 studies has found that several markers clearly differ between AD/MCI patients and healthy controls [8]. However, in this study we did not include healthy controls, but rather compared amyloid-positive patients to memory clinic patients without amyloid pathology, including patients with other dementias. Importantly, neuroinflammation is a shared feature of several neurodegenerative disorders including AD, frontotemporal dementia, Parkinson’s disease, dementia with Lewy bodies and vascular dementia [34, 48, 49], and several cytokines are similarly altered in AD versus frontotemporal dementia [34, 50, 51] or vascular dementia [49, 52]. The overlap between these different neurodegenerative disorders could explain why there was no difference in cytokine levels between the amyloid-positive and amyloid-negative groups in this study. Moreover, in this study the included patients were classified based exclusively on their amyloid status. Different approaches to classification and diagnoses are an important source of conflicting results in the literature [53]. Some research on the inflammatory markers in biomarker-defined groups has been restricted to amyloid-positive patients [14, 54]. Unfortunately, this precludes the comparison of amyloid-positive and amyloid-negative patients with cognitive disorders. However, one recent publication including both amyloid-positive and amyloid-negative patients demonstrated that the increased CSF cytokine levels were dependent on the abnormal tau and/or neurodegeneration biomarkers, while the cytokine levels were not increased in patients with isolated amyloid pathology [53]. Of note, in the current cohort the majority of patients in both amyloid groups had abnormal tau and/or neurodegeneration markers, which could explain the lack of group differences.

The current study was strengthened by the inclusion of a comprehensively characterised memory clinic cohort with extensive clinical and biological data, including multiple biomarker measurements and *APOE-*genotyping. By including other disease controls rather than cognitively healthy controls, this study can inform on the overlapping inflammatory mechanisms across different disorders and the use of inflammatory markers, or rather lack thereof, in differential diagnosis of memory clinic patients. A further strength is the application of the highly sensitive ThT-FCS assay. The ThT-FCS assay has the ultimate sensitivity and permits the detection of single aggregated particles in small sample volumes, within a wide range of molecular concentrations. This is achieved without the use of signal-amplification, protein separation or immune probes.

The current study has several limitations. First, the ThT-FCS method cannot identify the amino acid sequence of the polypeptides in the nanoplaques: ThT selectively binds aggregates with a β-sheet secondary structure. In addition to Aβ, several other proteins can aggregate to this structure and bind ThT [55, 56]. Therefore, the nanoplaques detected may not be composed exclusively of aggregated Aβ. Second, the sample size was limited, which restricted subgroup analyses. Third, this study did not include a healthy control group. Therefore, the relationship between nanoplaques and cytokines in the CSF of cognitively healthy individuals remains unknown; the level of cytokines in the absence of amyloid pathology may be below the detection threshold. Finally, the cross-sectional design of this study precluded the monitoring of the relationship between nanoplaques and cytokines over time. This is especially relevant given the apparent biphasic nature of cytokines in AD, and longitudinal studies are required.

Future research should assess whether the nanoplaques and cytokines are also associated in serum samples. Moreover, studies on microvascular pathology, a common feature of AD [57], are another avenue for future research: MIP-1α receptors are highly expressed on brain microvessels and it has been hypothesised that MIP-1α binding could affect angiogenesis and blood-brain barrier permeability [58–60]. IL-8 has also been linked to angiogenesis and blood-brain barrier dysfunction [61, 62]. As larger Aβ aggregates have been shown to contribute to neuroinflammation by compromising the blood-brain barrier [16], the relationship of markers of blood-brain barrier integrity with nanoplaque levels and cytokines in CSF and serum would be of interest.

## Conclusions

In conclusion, this study suggests a negative relationship between CSF amyloidogenic nanoplaques and cytokines MIP-1β, MIP-1α and IL-8. This finding contrasts with in vitro studies that have shown increased inflammation in response to aggregated amyloid from the CSF. However, the direction of this association is in line with recent evidence suggesting a protective role of the upregulation of certain cytokines. The relationship between nanoplaques and inflammatory markers should be further explored across the different stages of the AD pathophysiological process, in order to inform on the dynamics between aggregated amyloid and inflammation through the disease.

## Data Availability

Legal restrictions, imposed by the registry owners and the ethical committee, prevented us from publicly sharing the de-identified dataset due to sensitive patient information. The clinical data may be requested from the Norwegian Registry of Persons Assessed for Cognitive Symptoms at e-mail: post@aldringoghelse.no. The results of the ThT-FCS analysis and the cytokine levels are available upon reasonable request to the authors. All data availability is dependent on the approval from the REC South East, contact at e-mail: post@helseforskning.etikkom.no.

## Abbreviations

Aβ: Amyloid-β
AD: Alzheimer’s disease
*APOE*: Apolipoprotein E
CDR: Clinical Dementia Rating scale
CDR-SOB: Clinical Dementia Rating scale sum of boxes
CI: Confidence interval
CSF: Cerebrospinal fluid
FCS: Fluorescence Correlation Spectroscopy
*f*seo: Frequency of single event occurrence per hour
G-CSF: Granulocyte colony-stimulating factor
IL: Interleukin
IP: Interferon gamma-induced protein
LLOQ: Lower limit of quantification
MCI: Mild cognitive impairment
MCP: Monocyte chemoattractant protein
MIP: Macrophage inflammatory protein
MMSE: Mini-Mental State Examination
MRI: Magnetic resonance imaging
NIA-AA: National Institute of Aging and Alzheimer’s Association
NorCog: Norwegian Registry of Persons Assessed for Cognitive Symptoms
PET: Positron emission tomography
P-tau: Phosphorylated tau
Q: Quartile
SCD: Subjective cognitive decline
ThT: Thioflavin-T
ThT-FCS: Thioflavin-T-Fluorescence Correlation Spectroscopy
T-tau: Total tau
